# Identification of Candidate Genes for Low Phosphorus Tolerance in Maize Seedling Stage Based on GWAS and Transcriptome

**DOI:** 10.3390/plants14182836

**Published:** 2025-09-11

**Authors:** Xiaojia Hao, Gonxin Lei, Zhiming Zhong, Zelong Zhuang, Jianwen Bian, Lei Zhang, Wanling Ta, Zhenping Ren, Yunling Peng

**Affiliations:** 1 College of Agronomy, Gansu Agricultural University, Lanzhou 730070, China; haoxj772@163.com (X.H.); leigx2306@163.com (G.L.); zhuangzl3314@163.com (Z.Z.); bjwen1018@163.com (J.B.); 18919106150@163.com (L.Z.); kellytwl@163.com (W.T.); renzp1003@163.com (Z.R.); 2 Gansu Provincial Key Laboratory of Aridland Crop Science, Gansu Agricultural University, Lanzhou 730070, China; 3 Institute of Geographic Sciences and Natural Resources Research, Chinese Academy of Sciences, Beijing 100190, China; zhongzm@igsnrr.ac.cn

**Keywords:** candidate genes, differentially expressed genes (DEGs), genome-wide association study (GWAS), low phosphorus tolerance, maize seedling stage, transcriptome sequencing

## Abstract

Phosphorus is an essential nutrient for maize growth and development, and its deficiency can significantly inhibit plant growth, leading to reduced yield and quality. To elucidate the genetic mechanisms underlying low phosphorus tolerance in maize, this study utilized a panel of 257 maize inbred lines and conducted controlled experiments under low phosphorus (LP) and normal phosphorus (CK) conditions in artificial climate chambers. Through genome-wide association study (GWAS), a total of 46 SNP loci significantly associated with low phosphorus tolerance were detected, and 74 candidate genes were predicted. To further investigate, the low-phosphorus tolerant material CML422 and the phosphorus-sensitive material CIMBL90 were selected for transcriptome sequencing, which identified a total of 7232 differentially expressed genes (DEGs). KEGG enrichment analysis revealed that these genes were significantly enriched in key pathways such as plant hormone signal transduction, MAPK signaling pathway, and starch and sucrose metabolism, suggesting that maize responds to low phosphorus stress through the coordinated regulation of multiple pathways. By integrating GWAS and transcriptome data, 18 co-localized genes were screened, ultimately identifying 10 candidate genes closely associated with low phosphorus tolerance during the maize seedling stage, which are potentially involved in regulating growth and development under phosphorus stress. This study preliminarily elucidates the molecular mechanisms underlying low phosphorus tolerance in maize through multi-omics analysis, providing both a theoretical basis and genetic resources for breeding new maize varieties with high phosphorus use efficiency.

## 1. Introduction

Maize is a versatile crop widely utilized globally for food, feed, and fuel production. As one of the three major staple crops with the largest cultivation area worldwide, its demand is growing rapidly [1]. Maize is frequently adversely affected by various abiotic stresses, with low phosphorus stress being particularly prominent in tropical and subtropical regions [2]. Research indicates that approximately 57% of the natural terrestrial surface exhibits soil microbial activity limited by phosphorus, 39% is constrained by nitrogen, and 21% of soils experience co-limitation by both nitrogen and phosphorus [3]. Phosphorus (P) is an essential nutrient for plants and plays an irreplaceable role in energy metabolism and protein synthesis. As a key structural component of nucleic acids, phospholipids, and ATP, phosphorus significantly influences crop growth, development, and yield formation [4]. Phosphorus in the soil exists predominantly in the form of inorganic phosphates, such as H_2_PO_4_^−^ and HPO_4_^2−^. Its concentration is typically low, ranging from 0.1 to 10 μM [5]. The concentration of plant-available phosphate (soluble phosphate) is even lower and falls far below the level required for optimal growth [6]. Phosphorus is abundant in soil but readily forms insoluble compounds with Fe^3+^, Al^3+^, Ca^2+^, and Mg^2+^. Due to the prevalence of these bound forms, the relative availability of phosphorus to plants remains low [7]. The overapplication of phosphate fertilizers and their runoff into rivers result in low phosphorus use efficiency, adversely affecting the environment and ecosystems [8]. Moreover, long-term excessive application of phosphate fertilizers can lead to increased levels of cadmium and arsenic, potentially elevating carcinogenic risks [9]. Therefore, improving the absorption and utilization efficiency of phosphorus in maize is conducive to enhancing both yield and quality.

Plants have evolved a range of morphological, physiological, biochemical, and molecular strategies to cope with the stress induced by low phosphorus environments [10]. Plants respond to low phosphorus stress by altering root morphological traits, including root hair length and density, number of lateral roots, root-to-shoot ratio (RMR), total root surface area (TSA), and average root diameter (AVD) [11]. Modifications in root architecture, along with the exudation of phosphorus (P) and release of organic acids, enhance phosphorus uptake in plants [12]. Studies have shown that overexpression of AtPAP2 in Arabidopsis significantly enhances the production of sucrose and ATP in leaves [13]. Furthermore, proteins such as AtPAP18 and AtPAP25 have been demonstrated to be involved in the response to low phosphorus stress in Arabidopsis [14,15]. It is noteworthy that the central regulator of phosphorus response, PHR (Phosphate Starvation Response), has been identified in multiple crop species [16]. Phosphate Starvation Response 1 (AtPHR1) in Arabidopsis and OsPHR2, which encodes a MYB transcription factor in rice, are central regulators involved in the phosphorus signaling pathway [17]. AtPHR1 regulates a set of phosphate starvation-induced (PSI) genes by binding to the P1BS cis-element in their promoter regions [18]. Recent studies have revealed that the long non-coding RNA PILNCR2 modulates maize growth and phosphate uptake under phosphate stress by interfering with miR399-mediated cleavage of PHT transcripts [19]. In maize, genes including ZmPHT1;2, ZmPHT1;4, ZmPHT1;6, ZmPHT1;7, ZmPHT1;9, and ZmPHT1;11 are upregulated by arbuscular mycorrhizal fungi (AMF), suggesting their potential involvement in AMF-induced phosphorus uptake and/or transport processes [20].

Genome-wide association studies (GWAS) have become a powerful tool for uncovering complex genotype-phenotype associations across diverse species [21]. GWAS is widely used in various crops such as maize, wheat, rice, rapeseed, and cotton [21,22,23,24,25]. Under different phosphorus treatment levels, a genome-wide association study (GWAS) was conducted on 359 maize inbred lines for traits including biomass, grain yield, and phosphorus use efficiency (PUE). A total of 150 candidate genes were identified, among which seven genes associated with phosphate transport or stress response were selected for further molecular functional validation and characterization [26]. Under both field and greenhouse conditions, a genome-wide association study (GWAS) was performed on 132 maize inbred lines under low-phosphorus and normal-phosphorus treatments to analyze phosphorus use efficiency (PUE). The study identified 306 quantitative trait nucleotides (QTNs) associated with PUE and 186 potential candidate genes, which are primarily involved in transcriptional regulators, transporters, and proteins with transfer activity [27]. RNA sequencing (RNA-seq) can be used to describe or identify genes and obtain accurate transcriptional level information. In addition, it is also a valuable tool for analyzing gene regulatory networks by identifying differentially expressed genes (DEGs). Weighted gene co-expression network analysis (WGCNA) is one of the most popular methods for discovering hub factors controlling traits, which allows the discovery of core gene networks by analyzing gene expression patterns in RNA-seq data [28,29]. A comparative transcriptome analysis under phosphorus-deficient and normal phosphorus supply conditions revealed distinct expression patterns across various developmental stages and tissues in maize, and identified multiple candidate genes involved in the phosphorus stress response, including ZmPHT1-3, ZmPHT1-4, ZmPHT1-10, and ZmPHO1-H3 [30]. Furthermore, under low-phosphate (Pi) conditions, the expression of phosphate transporters and phosphatases was specifically induced in roots at both the transcript and protein levels. Concurrently, a significant increase was detected in the mRNA and corresponding protein levels of two purple acid phosphatases (PAPs) and one UDP-sulfoquinovose synthase (SQD) in the root system [31]. The combination of GWAS (genome-wide association analysis), WGCNA (weighted gene co-expression network analysis), and DEGs (differentially expressed genes) can more comprehensively explore the genes related to traits and their functional networks. For instance, one study utilized a multi-environment trial with 307 diverse maize inbred lines to investigate the relationships among mature grain phosphorus content, starch content, and hundred-kernel weight (HKW). It revealed that Zm00001d052392 was significantly correlated with phosphorus content and HKW, exhibiting high expression in SCML0849 but negligible expression in ZNC442 [32]. However, there remains a limited number of studies reporting on the molecular mechanisms underlying low phosphorus tolerance in maize. To elucidate the genetic mechanisms of low phosphorus tolerance during the maize seedling stage and enhance phosphorus use efficiency, further in-depth research is necessary. This study utilized a panel of 257 maize inbred lines for genome-wide association study (GWAS) analysis and further conducted RNA-seq analysis on low phosphorus-tolerant and phosphorus-sensitive materials selected from the panel. Finally, an integrated analysis based on GWAS and RNA-seq was conducted to further elucidate the genetic mechanisms underlying low phosphorus tolerance in maize.

## 2. Results

### 2.1. Phenotypic Analysis of Maize Under Phosphorus Stress at Seedling Stage

**Figure 1:**
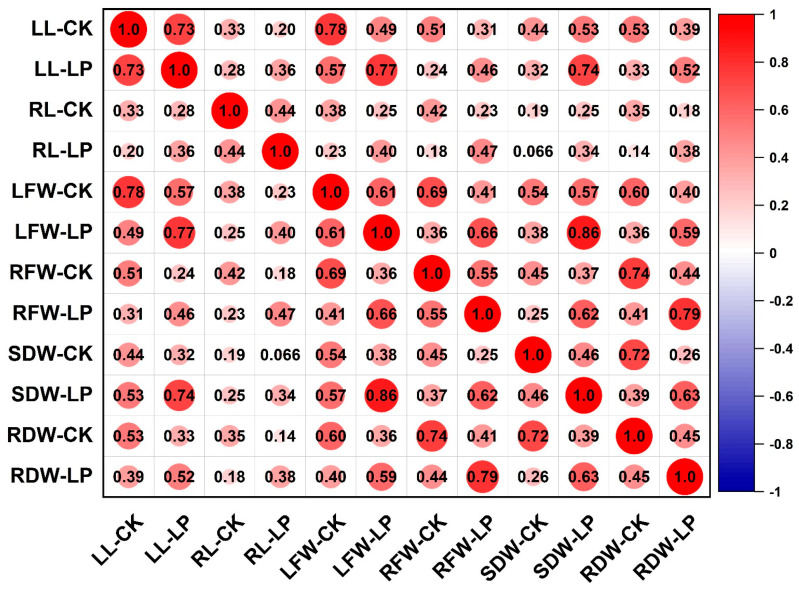
Pearson correlation analysis of various traits under CK (normal conditions) and LP (low phosphorus conditions). LL, RL, LFW, RFW, SDW, and RDW represent shoot length, root length, shoot fresh weight, root fresh weight, shoot dry weight, and root dry weight, respectively. The color and size of the circles reflect the value of the correlation coefficient.

**Figure 2:**
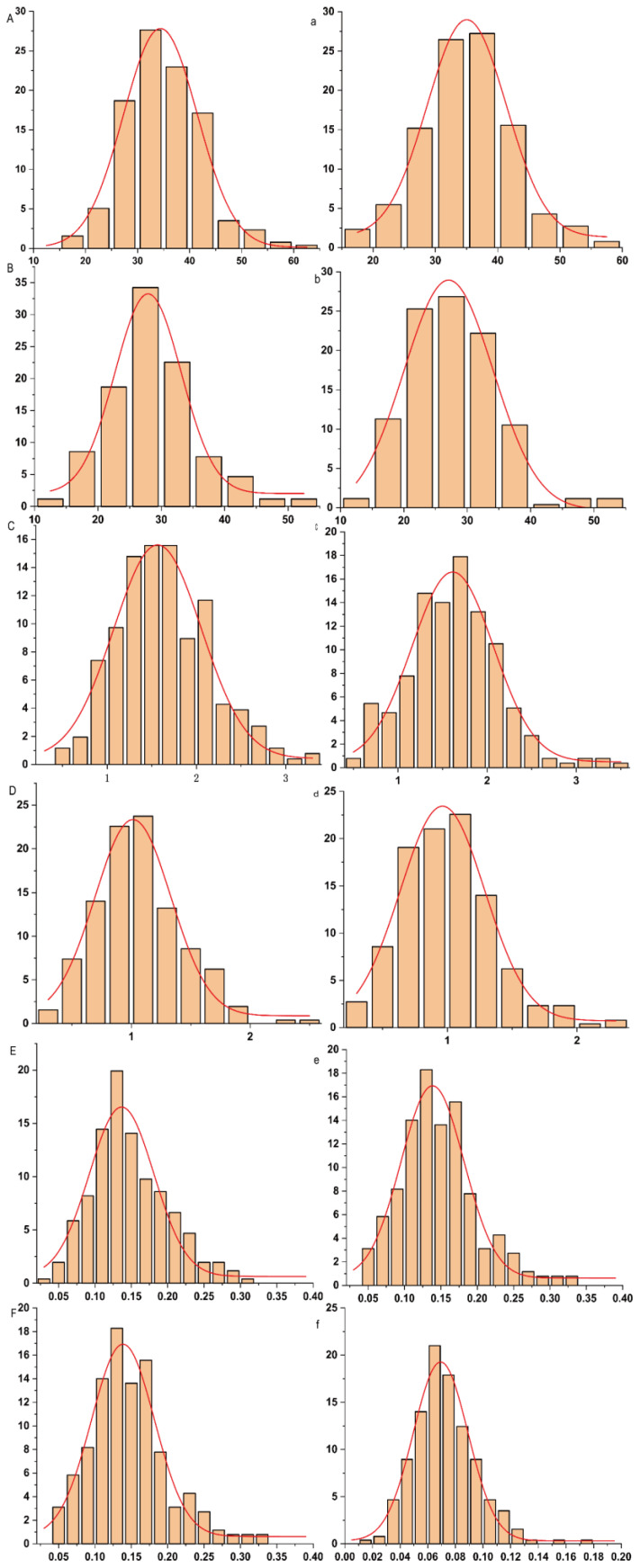
Frequency distribution of traits related to low phosphorus tolerance at maize seedling stage. Note: (**A**, **B**, **C**, **D**, **E**, **F**) represent shoot length (SL), root length (RL), shoot fresh weight (SFW), shoot dry weight (SDW), root fresh weight (RFW), and root dry weight (RDW) under normal (CK) conditions, respectively; (**a**, **b**, **c**, **d**, **e**, **f**) represent shoot length (SL), root length (RL), shoot fresh weight (SFW), shoot dry weight (SDW), root fresh weight (RFW), and root dry weight (RDW) under low phosphorus (LP) conditions, respectively.

Phenotypic analysis of 257 maize inbred lines under control and low-phosphorus conditions (Table 1) revealed continuous variation patterns across all measured traits, including leaf length (LL), root length (RL), leaf fresh weight (LFW), root fresh weight (RFW), leaf dry weight (LDW), and root dry weight (RDW). Under control conditions (CK), the coefficients of variation for each trait were 20.66%, 24.33%, 32.03%, 34.43%, 60.97%, and 35.49%, respectively. Under low phosphorus treatment (LP), the values were 20.81%, 25.27%, 31.96%, 36.27%, 37.53%, and 33.93%, respectively. Among these, leaf dry weight exhibited the highest coefficient of variation under both conditions (CK: 60.97%; LP: 37.53%), whereas leaf length showed the lowest (CK: 20.66%; LP: 20.81%). Pearson correlation analysis of the measured traits under both LP and CK treatments (Figure 1 and Supplementary Table S1) revealed significant correlations among most traits across different phosphorus conditions, with correlation coefficients ranging from 0.14 to 0.86. The only exception was the non-significant correlation between shoot dry weight under CK (SDW-CK) and root length under LP (RL-LP). These results indicate that traits associated with low phosphorus tolerance in maize interact and influence each other, collectively regulating plant growth under phosphorus stress. Under low phosphorus conditions, leaf length and root dry weight showed almost no significant changes, whereas root length (RL), leaf fresh weight (LFW), root fresh weight (RFW), and shoot dry weight (SDW) all decreased, by 3.28%, 0.61%, 5.66%, and 6.67%, respectively. Analysis of variance indicated that leaf length (LL), root length (RL), leaf fresh weight (LFW), root fresh weight (RFW), shoot dry weight (SDW), and root dry weight (RDW) were significantly influenced by genotype, environment, and genotype-by-environment interaction. The broad-sense heritability ranged from 0.62 to 0.89, with RL exhibiting the lowest heritability (0.62) and SDW the highest (0.89), demonstrating that these traits are primarily governed by genetic factors. Furthermore, under both phosphorus treatments, all investigated traits exhibited a normal or approximately normal distribution (Figure 2), demonstrating typical quantitative genetic characteristics and suitability for association analysis.

### 2.2. GWAS Analysis of Low Phosphorus Tolerance Traits in Maize Seedling Stage

**Figure 3:**
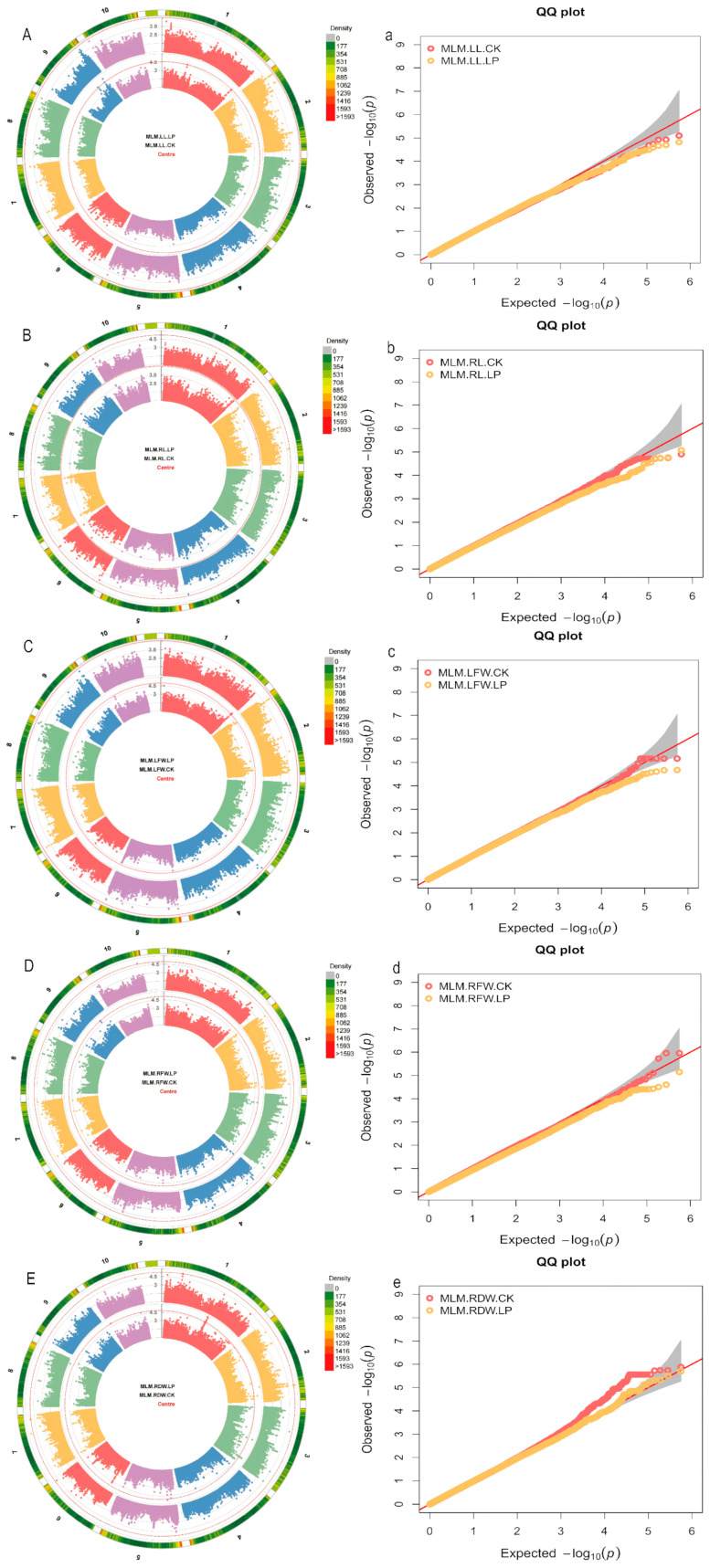
Manhattan and Q-Q plots of traits related to low phosphorus stress in maize under the MLM model. (**A**–**E**) Manhattan plots for shoot length (LL), root length (RL), shoot fresh weight (LFW), root fresh weight (RFW), and root dry weight (RDW) based on the MLM model; (**a**–**e**) Q-Q plots for shoot length (LL), root length (RL), shoot fresh weight (LFW), root fresh weight (RFW), and root dry weight (RDW) based on the MLM model.

In this study, a mixed linear model (MLM) was employed to perform genome-wide association studies (GWAS) for each trait (Figure 3). In the GWAS analysis of 5 related traits, a total of 46 significant SNPs were identified using a significance threshold of *p* = 1 × 10^−5^ (Supplementary Table S2). Under normal (CK) conditions, significant loci were detected for the following traits: one locus for leaf length (LL), seven for leaf fresh weight (LFW), four for root fresh weight (RFW), and twenty-five for root dry weight (RDW). Under low phosphorus (LP) conditions, significant loci included: one for root length (RL), one for root fresh weight (RFW), and seven for root dry weight (RDW). It is noteworthy that multiple adjacent loci associated with the same trait are located on the same chromosome (e.g., chr1.S_297373478, chr2.S_183296885, chr1.S_170578442, chr5.S_31179619, chr10.S_5223950, chr7.S_162003719). Additionally, under normal (CK) conditions, the locus chr2.S_183296885 was associated with both root fresh weight (RFW) and root dry weight (RDW), with adjacent loci also identified in the same region.

### 2.3. Gene Function Annotation in Candidate Intervals

Gene identification was performed within a 200 kb region based on single nucleotide polymorphisms (SNPs) present in the population. The B73_RefGen_v3 reference genome was searched in MaizeGDB (https://maizegdb.org/), using a 200 kb interval upstream and downstream of each SNP as the candidate region. A total of 74 genes were identified (Supplementary Table S3). Under normal (CK) conditions, four genes were associated with leaf length (LL), four with leaf fresh weight (LFW), nine with root fresh weight (RFW), and twenty-eight with root dry weight (RDW). Under low phosphorus (LP) conditions, six genes were linked to root length (RL), four to root fresh weight (RFW), and nineteen to root dry weight (RDW).

### 2.4. Transcriptome Analysis of Low Phosphorus Tolerance in Maize Seedling Stage

#### 2.4.1. Transcriptome Sequencing Results and Quality Assessment and Genome Comparison Rate

RNA-seq sequencing of 24 samples yielded a total of 160.95 Gb of Clean Data. Each sample produced 6.00 Gb of Clean Data, with GC content ranging from 52.68% to 56.13%, and Q20 and Q30 values reaching 96.72% and 92.2%, respectively (Supplementary Table S4). The clean reads from maize were aligned to the maize reference genome, resulting in alignment rates ranging from 80.23% to 92.77% across samples (Supplementary Table S5).

#### 2.4.2. Differential Gene Analysis

**Figure 4:**
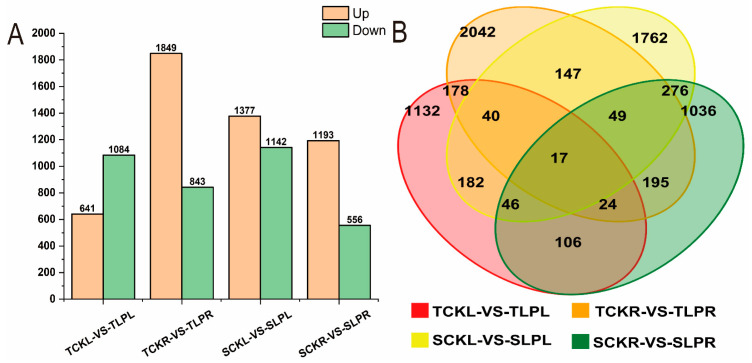
Statistics of differentially expressed gene counts in maize seedlings under phosphorus stress. (**A**): Bar plot of the number of differentially expressed genes across comparison groups; (**B**): Venn diagram of DEGs among different control groups.

Bar and Venn diagrams illustrated the numbers of up- and down-regulated differentially expressed genes (DEGs) in maize seedlings under phosphorus stress, based on the criteria of a fold change (FC) ≥ 1.5 and a *p*-value ≤ 0.05 (Figure 4 and Supplementary Table S6). Compared to the control, the comparison TCKL-vs-TLPL identified 1725 differentially expressed genes (DEGs), including 641 up-regulated and 1084 down-regulated genes; the comparison TCKR-vs-TLPR identified 2692 DEGs, consisting of 1849 up-regulated and 843 down-regulated genes. In the SCKL-vs-SLPL comparison, 2519 differentially expressed genes (DEGs) were identified, comprising 1377 up-regulated and 1142 down-regulated genes; in the SCKR-vs-SLPR comparison, 1749 DEGs were detected, consisting of 1193 up-regulated and 556 down-regulated genes. The Venn diagram revealed 17 genes that were co-expressed in both low phosphorus-tolerant and phosphorus-sensitive materials. The results indicate that under phosphorus stress during the maize seedling stage, low phosphorus-tolerant roots respond by upregulating a greater number of differentially expressed genes, whereas phosphorus-sensitive materials respond through more up-regulated genes in the leaves.

#### 2.4.3. GO Enrichment Analysis of DEGs in Maize Seedlings Under Phosphorus Stress

**Figure 5:**
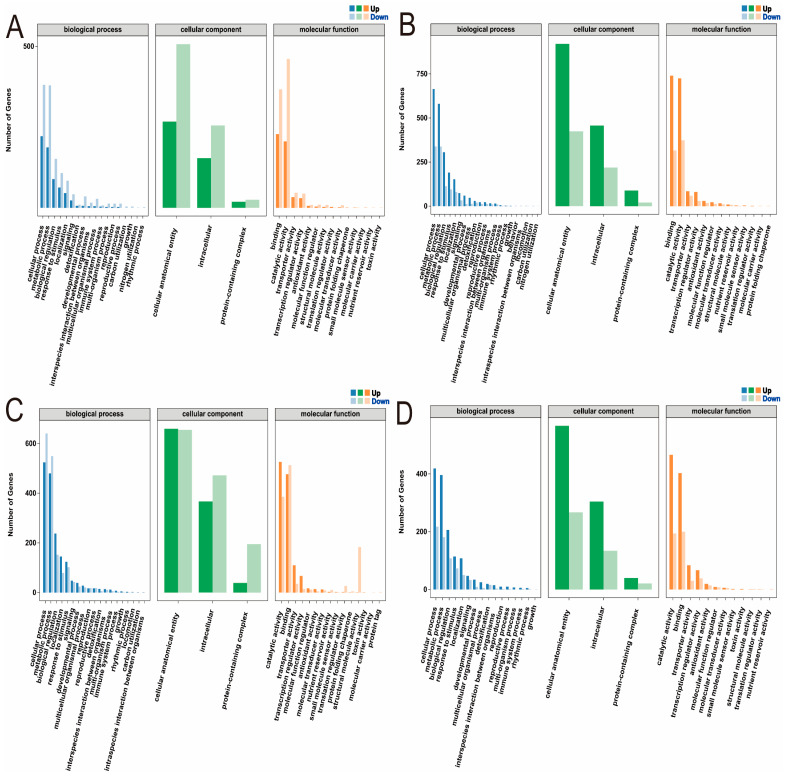
GO annotation of DEGs in maize seedlings under phosphorus stress. (**A**): TCKL-vs-TLPL; (**B**): TCKR-vs-TLPR; (**C**): SCKL-vs-SLPL; (**D**): SCKR-vs-SLPR.

GO functional enrichment analysis was performed on the differentially expressed genes in the leaves and roots of low phosphorus-tolerant (CML422) and phosphorus-sensitive (CIMBL90) materials, respectively, covering biological process, cellular component, and molecular function (Supplementary Table S7). In the TCKL-vs-TLPL comparison, differentially expressed genes in the biological process category were primarily enriched in cellular processes, metabolic processes, and biological regulation; those in the cellular component category were mainly enriched in cellular anatomical entities, intracellular regions, and protein-containing complexes; and genes in the molecular function category were predominantly enriched in binding, catalytic activity, and transporter activity (Figure 5A). In the TCKR-vs-TLPR comparison, differentially expressed genes in the biological process category were predominantly enriched in cellular processes, metabolic processes, and biological regulation; those in the cellular component category were mainly enriched in cellular anatomical entities, intracellular regions, and protein-containing complexes; and genes in the molecular function category were primarily enriched in binding, catalytic activity, and transporter activity (Figure 5B). In the SCKL-vs-SLPL comparison, differentially expressed genes in the biological process category were predominantly enriched in cellular processes, metabolic processes, and biological regulation; those in the cellular component category were mainly enriched in cellular anatomical entities, intracellular regions, and protein-containing complexes; and genes in the molecular function category were primarily enriched in catalytic activity, binding, and transporter activity (Figure 5C). In the SCKR-vs-SLPR comparison, differentially expressed genes in the biological process category were predominantly enriched in cellular processes, metabolic processes, and biological regulation; those in the cellular component category were mainly enriched in cellular anatomical entities, intracellular regions, and protein-containing complexes; and genes in the molecular function category were primarily enriched in catalytic activity, binding, and transporter activity (Figure 5D).

The enriched terms were largely consistent across the four comparison groups, and a three-level analysis was conducted for each term. In the TCKL-vs-TLPL comparison, the biological process category showed enrichment in positive regulation of transcription, DNA-templated, microtubule-based processes, response to water deprivation, metal ion transport, regulation of defense response, response to wounding, and fatty acid biosynthetic processes. The molecular function category was enriched in metal ion binding, DNA-binding transcription factor activity, sequence-specific DNA binding, and heme binding. The cellular component category was enriched in integral component of membrane, plasma membrane, film, and cytoplasm (Supplementary Figure S1). In the TCKR-vs-TLPR comparison, the biological process category was enriched in carbohydrate metabolic process, antioxidant response, and hydrogen peroxide catabolic process; the molecular function category was enriched in metal ion binding and heme binding; and the cellular component category was enriched in integral component of membrane (Supplementary Figure S2). In the SCKL-vs-SLPL comparison, the biological process category was enriched in translation; the molecular function category was enriched in structural constituent of ribosome; and the cellular component category was enriched in cytosolic large ribosomal subunit and ribosome (Supplementary Figure S3). In the SCKR-vs-SLPR comparison, the biological process category was enriched in hydrogen peroxide catabolic process and antioxidant response; the molecular function category was enriched in DNA-binding transcription factor activity and heme binding; and the cellular component category was enriched in integral component of membrane, nucleus, and plasma membrane (Supplementary Figure S4).

#### 2.4.4. KEGG Enrichment Analysis of DEGs in Maize Seedlings Under Phosphorus Stress

**Figure 6:**
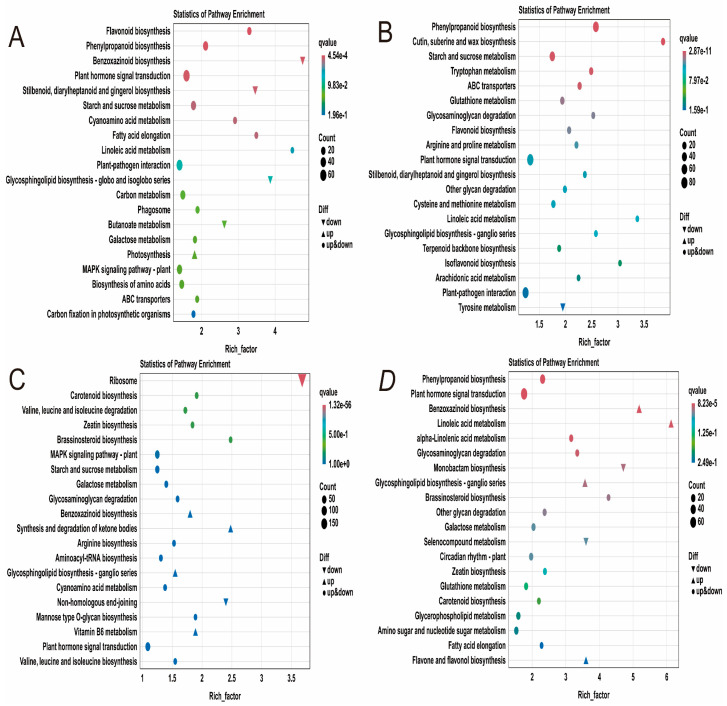
KEGG annotation of DEGs in maize seedlings under phosphorus stress. (**A**): TCKL-vs-TLPL; (**B**): TCKR-vs-TLPR; (**C**): SCKL-vs-SLPL; (**D**): SCKR-vs-SLPR.

To further investigate the response mechanisms of maize seedlings to phosphorus stress, KEGG functional annotation analysis was performed on the identified differentially expressed genes (Supplementary Table S8). The results revealed that six metabolic pathways were significantly enriched in the TCKL-vs-TLPL comparison, including plant hormone signal transduction, plant-pathogen interaction, the MAPK signaling pathway in plants, phenylpropanoid biosynthesis, starch and sucrose metabolism, as well as carbon metabolism and amino acid biosynthesis (Figure 6A). In the TCKR-vs-TLPR comparison, four metabolic pathways were significantly enriched: plant hormone signal transduction, plant-pathogen interaction, phenylpropanoid biosynthesis, and starch and sucrose metabolism (Figure 6B). In the SCKL-vs-SLPL comparison, five metabolic pathways were significantly enriched, including ribosome, plant hormone signal transduction, plant-pathogen interaction, the MAPK signaling pathway in plants, and starch and sucrose metabolism (Figure 6C). In the SCKL-vs-SLPL comparison, three metabolic pathways were significantly enriched: plant hormone signal transduction, phenylpropanoid biosynthesis, and amino sugar and nucleotide sugar metabolism (Figure 6D). The results indicate that under phosphorus stress, maize leaves primarily respond through plant hormone signal transduction, plant-pathogen interaction, the MAPK signaling pathway in plants, and starch and sucrose metabolism. In contrast, under low phosphorus stress, the roots mainly rely on plant hormone signal transduction and phenylpropanoid biosynthesis to cope with the stress.

#### 2.4.5. WGCNA Analysis of Maize Seedlings Under Phosphorus Stress

**Figure 7:**
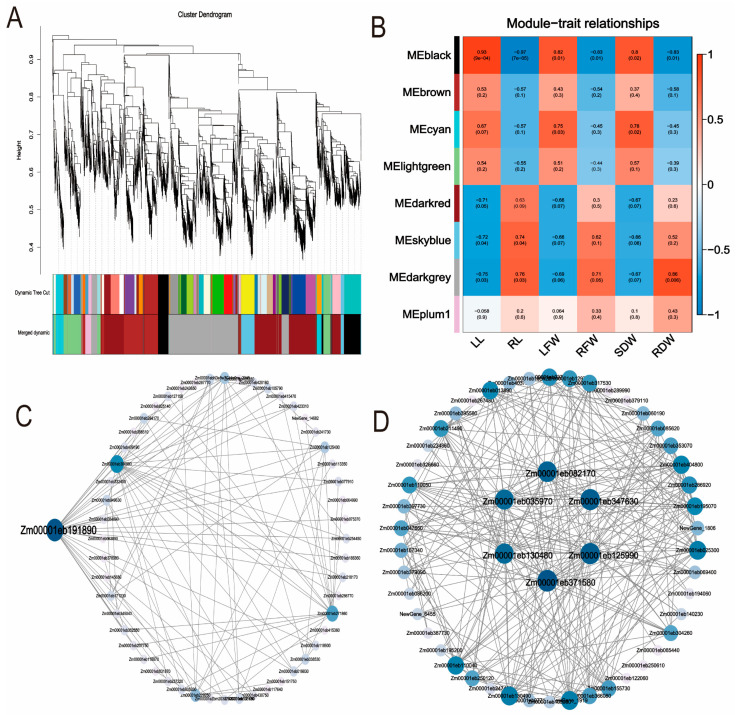
Construction of modules based on WGCNA. (**A**): Gene network modules; (**B**): Module–trait correlation heatmap; (**C**): Hub gene interaction network analysis in the MEblack module; (**D**): Hub gene interaction network analysis in the MEdarkgrey module.

In this study, a gene co-expression network was constructed using 24 samples. After removing genes with FPKM values less than 1, a total of 5016 differentially expressed genes were retained, and 9 co-expression modules were identified (Figure 7A). Analysis revealed significant correlations between LL and the MEblack module, RL and the MEblue module, LFW and the MEskyblue module, RDW and the MEdarkgrey module, as well as SDW and the MEcyan module (Figure 7B). Furthermore, co-expression network analysis identified hub genes within the MEblack and MEdarkgrey modules (Figure 7C,D). Specifically, the MEblack module contained the gene Zm00001eb191890, while the MEdarkgrey module included Zm00001eb082170, Zm00001eb035970, Zm00001eb371580, Zm00001eb130480, Zm00001eb125990, and Zm00001eb347630. Based on gene annotation information, it is hypothesized that starch synthase, cell wall-associated proteins, ABA-induced proteins, and pectin catabolic processes may play important roles in the regulatory mechanisms of maize response to phosphorus stress (Supplementary Table S9).

### 2.5. Integration Analysis of Candidate Gene Results Under Phosphorus Stress in Maize Seedling Stage

**Figure 8:**
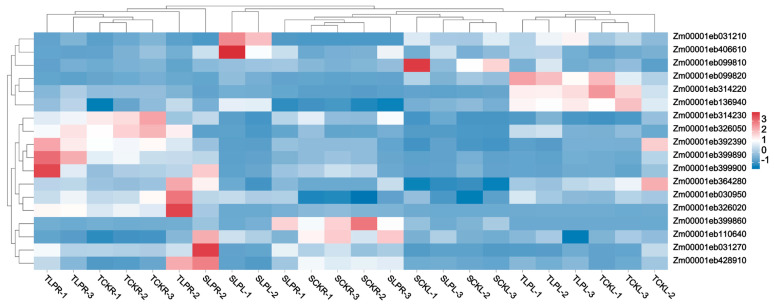
Heatmap of gene expression levels co-identified by GWAS and transcriptome analysis.

To identify candidate genes for low phosphorus tolerance, we selected differentially expressed genes (DEGs) from the four comparison groups under both normal and low phosphorus conditions as the gene set, based on transcriptome sequencing data. Through co-localization of GWAS and transcriptome analyses, we identified 18 candidate genes (Figure 8 and Supplementary Table S10). Subsequently, based on gene annotation information, we removed unannotated and potentially non-coding genes, ultimately identifying 10 candidate genes. These include: Zm00001eb030950, Zm00001eb031210, Zm00001eb031270, Zm00001eb099810, Zm00001eb099820, Zm00001eb136940, Zm00001eb314220, Zm00001eb314230, Zm00001eb364280, and Zm00001eb428910.

### 2.6. RT-qPCR Validation

**Figure 9:**
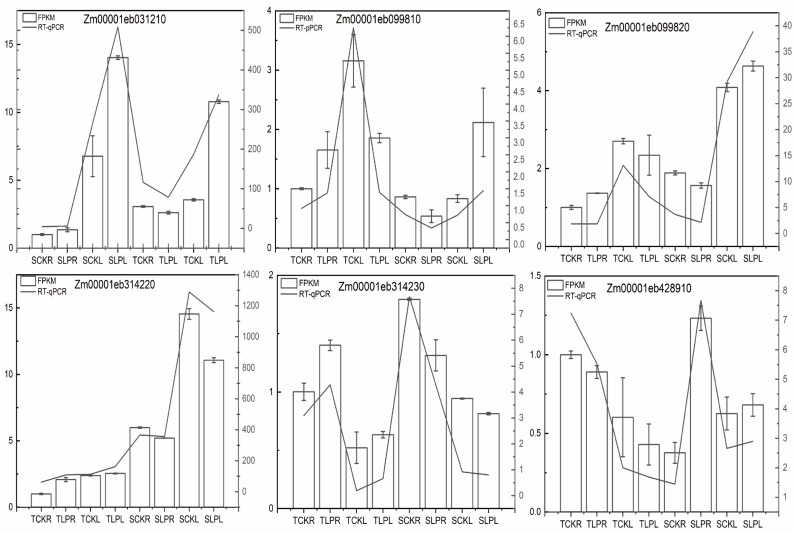
RT-qPCR was performed to verify the expression of six genes. The validation results of the transcriptome data were expressed using both the bar graph (RT-qPCR) and the line graph (RNA-Seq).

For the 18 genes co-localized by GWAS and transcriptome analysis, we selected six for qRT-PCR validation. As shown in Figure 9, the relative expression levels of Zm00001eb031210, Zm00001eb099810, Zm00001eb099820, Zm00001eb314220, Zm00001eb314230, and Zm00001eb428910 were consistent with the expression trends observed in the RNA-seq data.

## 3. Discussion

### 3.1. Analysis of Low Phosphorus Tolerance Phenotype of Maize

Under phosphorus-deficient conditions, maize plants exhibit significant growth inhibition, characterized by reduced plant height, decreased biomass accumulation, and a marked decline in photosynthetic efficiency [33]. Low phosphorus stress significantly alters maize root morphology, manifested by a reduction in root tip number, decreased root volume, and diminished surface area [34]. Under low phosphorus conditions, both the maximum shoot length (MSL) and maximum root length (MRL) of maize seedlings were lower than those under normal phosphorus conditions. Additionally, low phosphorus stress led to a significant reduction in total dry matter (TDM), shoot dry weight (SDW), and root dry weight (RDW) [35]. Under low phosphorus conditions, the low phosphorus-resistant line DSY2 exhibited longer root length, greater specific surface area and volume, as well as higher root activity and acid phosphatase activity compared to the low phosphorus-sensitive line DSY79 [36].

### 3.2. Genome-Wide Association Analysis of Low Phosphorus Tolerance in Maize Seedling Stage

During the maize seedling stage, GWAS analysis identified a substantial number of genes associated with low phosphorus stress. Among these, the gene Zm00001d022226 was significantly correlated with multiple phosphorus-related traits under low phosphorus conditions. Additionally, ZmSPX4.1 and ZmSPX2 were sharply up-regulated in response to low Pi stress across different lines or tissues [37]. Another study on GWAS of 13 traits in maize detected 551, 1140, and 1157 significant SNPs in the years 2012, 2016, and 2016, respectively, along with 23 shared candidate genes. Among these, seven overlapped with previously reported quantitative trait loci (QTLs) and genes related to low phosphorus stress. Furthermore, the Hap5 haplotype, which contains 12 favorable SNPs, was found to enhance root development and phosphorus uptake under low phosphorus stress [38]. In this study, we performed genome-wide association studies (GWAS) on 257 maize inbred lines for the following traits: shoot length (LL), root length (RL), shoot fresh weight (LFW), root fresh weight (RFW), and root dry weight (RDW). Through GWAS analysis, we identified 82 genes that either overlap with or are adjacent to SNPs associated with potential low phosphorus tolerance traits. Among these, Zm00001eb099830 encodes transcription factor MYB36. MYB36 functions in the outer endodermis during lateral root primordium (LRP) development through a mechanism analogous to regulating the root meristem proliferation/differentiation transition [39]. Zm00001eb406620 encodes probable purple acid phosphatase 20. Purple acid phosphatase (PAP) is an acid phosphatase that plays an important role in the uptake and metabolism of phosphorus (P). Low phosphorus increased the activity of acid phosphatase, promoted the transfer of soluble phosphorus from underground to aboveground, but also inhibited the growth and development of bamboo roots [40]. Zm00001eb064210 encodes the protein phosphatase 2A regulatory subunit B’. PP2A-B’ζ positively regulates seed germination and seedling development in plants. Mutants of PP2A-B’ζ exhibit heightened sensitivity to ethylene treatment. Furthermore, PP2A-B’ζ interacts with and stabilizes CTR1 (Constitutive Triple Response 1), a key enzyme in the ethylene signaling pathway. Similar to CTR1, PP2A-B’ζ negatively regulates ethylene signal transduction in plants [41]. Zm00001eb031250 encodes the protein ROOT HAIR DEFECTIVE 3 homolog 1. In poplar, PeRHD3 likely regulates the formation of adventitious roots, lateral roots, and root hair development by modulating anisotropic cell expansion [42]. Although there are relatively few reports directly linking the aforementioned genes to regulatory responses under phosphorus stress, phosphorus is an essential nutrient for plant growth, and its deficiency significantly impacts root development and function. Therefore, these genes may enhance maize adaptation to low phosphorus environments by modulating root growth and development or regulating the expression of related genes.

### 3.3. Differential Gene Expression and Weighted Gene Co-Expression Network Analysis

**Figure 10:**
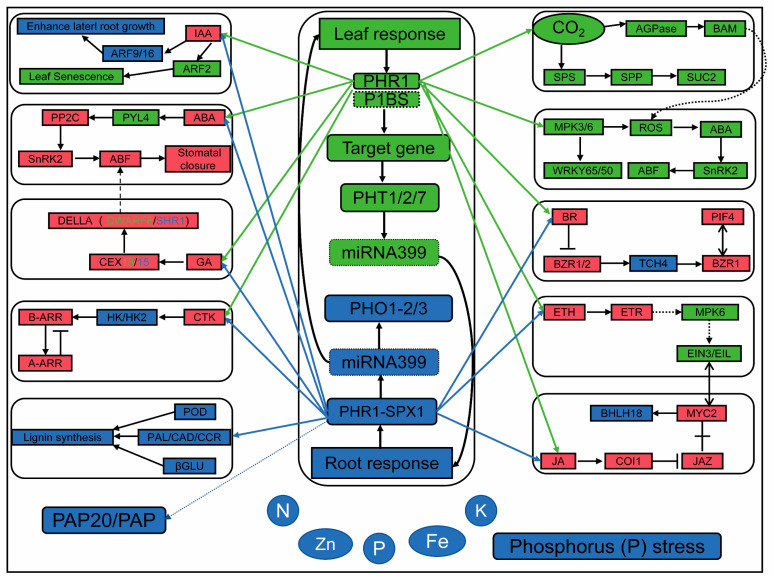
Main regulatory pathways of maize leaves and roots under phosphorus deficiency stress. The dotted line represents possibility and speculation; green represents leaf regulation; blue indicates root regulation; red indicates both.

The application of transcriptome sequencing (RNA-seq) for genome-wide gene expression studies not only enables effective dissection of gene regulatory networks underlying complex agronomic traits but also elucidates plant response mechanisms to environmental stress [43]. Phosphorus deficiency is one of the most severe abiotic stresses affecting maize. In-depth research on the molecular mechanisms underlying maize response to low phosphorus stress is of great significance for optimizing phosphorus fertilizer use, improving crop yield, and enhancing quality. In this study, we investigated the molecular mechanisms underlying the response of maize seedlings to low phosphorus stress through RNA-seq analysis. Transcriptomic results revealed that a greater number of differentially expressed genes were identified in low phosphorus-tolerant roots, whereas more were detected in phosphorus-sensitive leaves. Among the differentially expressed genes (DEGs), we observed significant changes in the expression levels of various transcription factors (TFs), phosphate transporter genes, and hormone-responsive genes, manifesting as either up- or down-regulation. These DEGs were primarily involved in biological processes such as plant nutrient metabolism, phosphate starvation response, phosphate transport, hormone response, and the regulation of oxidoreductase activity. Numerous genes from the WRKY, MYB, BZIP, and NAC transcription factor (TF) families have been reported to be involved in the regulation of phosphorus absorption [44]. The results indicate that under low phosphorus stress, the expression levels of PHR1 and PHT genes are significantly increased. The phosphate transporter 1 (PHT1) and its regulatory factor phosphate starvation response 1 (PHR1) collectively form the PHR1-PHT1 module, which plays a key role in maintaining phosphorus homeostasis across different organs. In soybean nodules, the presence of the GmPHR-GmPHT1 module promotes phosphorus accumulation and increases nodule size, but simultaneously reduces nodule number [45]. In this study, KEGG analysis identified plant hormone signal transduction and phenylpropanoid biosynthesis in maize roots, whereas plant hormone signal transduction, the MAPK signaling pathway in plants, and starch and sucrose metabolism were identified in the leaves (Figure 10). PHR, along with the long-distance signaling molecule microRNA399 (miR399) and the E2 ubiquitin-conjugating enzyme PHOSPHATE 2 (PHO2), forms a coordinated regulatory pathway in the phosphate starvation response [16]. PHR1 regulates the expression of AtPHO1. Under phosphorus-sufficient conditions, auxin and cytokinin upregulate the expression of AtPHO1; H1 and AtPHO1; H10, whereas their expression is downregulated in phosphorus-deficient plants. Additionally, abscisic acid suppresses the expression of AtPHO1 and AtPHO1;H1 under both phosphorus-sufficient and low-phosphorus conditions, but promotes increased expression of AtPHO1;H10 [46]. PHR1 exhibits cross-talk with ABA and sucrose. For example, PHR1 indirectly influences sucrose accumulation by regulating the OPEN STOMATA 1 (OST1) gene within the ABA signaling pathway [47]. PHR1 promotes starch accumulation by activating the expression of AGPase genes [48]. Both PHL1 and PHL4 are redundant to PHR1 and act equally in regulating leaf senescence, Pi-starvation-induced primary root growth inhibition, and anthocyanin accumulation in shoots [49]. Among plant hormones, for example, ARF2 restricts the expression levels of GRF transcription factors in proliferating tissues by repressing their expression. In Arabidopsis, ARF2 binds to conserved sequences and thereby suppresses GRF5 expression, limiting excessive leaf cell proliferation [50]. ARF9 and the brassinosteroid (BR) signaling pathway promote leaf thickness by inducing the transcription of genes associated with cell expansion, thereby enhancing drought tolerance in plants [51]. Furthermore, in maize, ARF16 collaborates with transcription factors such as MYB6 and bZIP1 to co-regulate the expression of phosphate starvation response genes, thereby influencing root growth and phosphorus uptake [52]. The interaction between BZR1 and PIF4 regulates a core transcriptional network, enabling plant growth to be coordinately modulated by steroid hormones and environmental signals [53]. The OsBZR1-SPX1/2 module balances plant growth and immunity in response to phosphorus (Pi) availability under varying phosphorus levels [54].

Through weighted gene co-expression network analysis (WGCNA), this study identified the MEblack module, which showed a significant correlation with leaf traits (correlation coefficient r = 0.93), and the MEdarkgrey module, which was significantly correlated with root traits (r = 0.83). The MEblack and MEdarkgrey modules contain seven hub genes. Among these, Zm00001eb082170 encodes protein NRT1/PTR FAMILY 4.4. In Arabidopsis, although NRT1.13 (NPF4.4) lacks nitrate transport capability, it is able to bind nitrate. Furthermore, NRT1.13 influences shoot architecture and flowering time by modulating internal nitrate levels near the xylem [55]. Zm00001eb371580 encodes an ABA-induced protein. Mutant plants lacking AIP1 exhibit reduced sensitivity to abscisic acid (ABA) and glucose during seed germination and the seedling stage. Studies indicate that AIP1 is involved in ABA-mediated cellular signal transduction and functions as a positive regulator of ABA [56]. Zm00001eb130480 encodes a pectinesterase. Exogenous ABA negatively regulates soluble phosphorus content in roots and shoots by reducing pectin levels. Simultaneously, it suppresses the phosphorus deficiency-induced increase in pectinesterase activity and the expression of the phosphate transporter gene OsPT6, thereby reducing phosphorus recycling from the cell wall and its translocation to shoots [57].

### 3.4. Candidate Genes and Molecular Regulatory Network of Low Phosphorus Tolerance in Maize

Through GWAS analysis, 259 candidate genes associated with low phosphorus stress were identified. These genes are primarily involved in transcriptional regulation, reactive oxygen species (ROS) scavenging, hormone regulation, and cell wall remodeling [58]. Transcriptome analysis further validated the GWAS results: among the 85 candidate genes, 45 were significantly regulated under low phosphorus conditions. The absence of the OsPSTOL1 gene did not exhibit significant differences under phosphorus deficiency, suggesting that other QTLs may contribute to rice adaptation to low phosphorus environments [59]. Another study, through the integration of multi-omics data, revealed that ZmG6PE modulates low phosphorus stress by regulating the expression of ZmSPX6 and ZmPHT1.13. ZmG6PE participates in the phosphate signaling pathway and influences maize yield-related traits by balancing carbohydrate homeostasis [60]. In this study, transcriptome sequencing analysis was used to compare differentially expressed genes (DEGs) between low phosphorus-tolerant and phosphorus-sensitive varieties under both low and normal phosphorus conditions. A total of 7232 DEGs were identified, which were selected as the gene set for phosphorus deficiency tolerance. This study identified 46 quantitative trait locus (QTL) regions through genome-wide association analysis, encompassing 73 genes. Based on gene annotation information, 43 relevant genes were selected. Integration of the phosphorus deficiency tolerance gene set with the annotation results led to the co-localization of 18 genes. After removing unannotated and putative non-coding genes, 10 candidate genes were ultimately identified. Among these, Zm00001eb099810 and Zm00001eb099820 encode glutamate receptor 2.8/2.9 (GLR), which functions as a critical calcium ion channel in plants and regulates calcium signal transduction. GLRs play important roles in various plant-specific physiological processes, including pollen tube growth, sexual reproduction, root meristem proliferation, internode cell elongation, stomatal regulation, as well as innate immunity and wound response [61]. Exogenous γ-aminobutyric acid (GABA) and Ca^2+^ can enhance stress resistance in peanut growth under phosphorus-deficient conditions [62]. Zm00001eb399860 encodes glutamine synthetase 3 (GS3). Glutamine synthetase is a key enzyme in nitrogen assimilation, catalyzing the combination of ammonium ions (NH_4+_) with glutamate to form glutamine. Consequently, this enzyme plays a crucial role in plant growth and productivity [63]. Under phosphorus-deficient conditions, the activity of glutamine synthetase (GS) in cyanobacteria is significantly reduced, leading to impaired nitrogen assimilation and consequently affecting cell survival [64]. Zm00001eb031210 encodes Basic leucine zipper 25 (bZIP25), which belongs to the basic leucine zipper (bZIP) transcription factor family. This family comprises evolutionarily conserved transcription factors (TFs) in eukaryotes. bZIP TFs are widely distributed in plants and play important roles in processes such as plant growth and development, photomorphogenesis, signal transduction, defense against pathogens, response to biotic and abiotic stresses, and secondary metabolism [65]. BZIP25 and BZIP53 play key roles in the development of epidermal cells in Arabidopsis leaves. These two transcription factors are highly expressed in pavement cells and early meristematic cells, and regulate epidermal cell differentiation through the jasmonic acid (JA) signaling pathway [66]. Zm00001eb136940 encodes the serine/threonine protein kinase EDR1. The ethylene-induced senescence phenotype mediated by EDR1 is suppressed by EIN2 mutation but not by mutations in SID2, PAD4, EDS1, or NPR1. EDR1 functions in the cross-talk between ethylene and salicylic acid signaling, influencing senescence and cell death [67]. Zm00001eb428910 encodes the protein argonaute 1B (ARGONAUTE 1B). ARGONAUTE (AGO) proteins serve as core components of the RNA-induced silencing complex (RISC). They bind to small non-coding RNAs (sRNAs) and target complementary RNAs, thereby triggering translational repression or initiating endonucleolytic cleavage pathways [68]. Overexpression of OsAGO1b resulted in proximal leaf curling and induced a range of abnormal phenotypes, such as reduced tiller number and plant height. In OsAGO1b knockdown lines, leaves appeared largely normal, but seed setting rate was significantly decreased, accompanied by disordered anther patterning and reduced pollen fertility [69]. The Zm00001eb314230 gene encodes the GAMETE EXPRESSED 1 (GEX1) protein, a key membrane protein highly conserved in flowering plants that is primarily involved in nuclear fusion and embryonic development. In cotton, the GEX1 gene is expressed across various developmental stages and multiple tissues. Studies have shown that loss of GhGEX1 significantly suppresses differentially expressed genes (DEGs) related to reproductive and membrane-associated processes in pollen, thereby impairing normal pollen physiological function [70].

## 4. Materials and Methods

### 4.1. Experimental Materials and Culture Conditions

This study utilized 257 maize inbred lines derived from a set of 368 accessions introduced by the Institute of Crop Science, Chinese Academy of Agricultural Sciences, from the International Maize and Wheat Improvement Center (CIMMYT), Mexico. The seed propagation was carried out in 2020 at the maize breeding experimental field of Huangyang Experimental Station (37.97° N, 102.63° E) in Wuwei City, Gansu Province, by the Maize Research Group of Gansu Agricultural University (Supplementary Table S11) [71,72]. This experiment was conducted under normal (CK) and low phosphorus (LP) conditions. The CK nutrient solution consisted of 2 mmol/L KH_2_PO_4_, 2 mmol/L Ca(NO_3_)_2_·4H_2_O, 0.1 mmol/L KCl, 1.25 mmol/L NH_4_NO_3_, 0.65 mmol/L K_2_SO_4_, 0.65 mmol/L MgSO_4_·7H_2_O, 0.01 mmol/L H_3_BO_3_, 5 nmol/L (NH_4_)_6_Mo_7_O_24_, 1 μmol/L MnSO_4_, 1 μmol/L CuSO_4_·5H_2_O, 1 μmol/L ZnSO_4_·7H_2_O, and 0.1 mmol/L Fe-EDTA. The LP nutrient solution replaced KH_2_PO_4_ with KCl while keeping all other components identical to CK. Uniform and plump seeds were selected, sterilized with 0.5% NaClO solution for 30 min, rinsed 3–5 times with distilled water, and then soaked in distilled water for approximately 12 h. After thoroughly mixing distilled water with vermiculite, maize seeds were sown in pots with a diameter of 10 cm. The pots were then placed in an artificial climate chamber set to a daytime temperature of 24 °C, nighttime temperature of 22 °C, relative humidity of 60–80%, 12 h of light, and a light intensity of 600 μmol/m^2^/s. Each treatment included three replicates, with each replicate consisting of four seedlings. Irrigation was performed every two days with 50 mL of the corresponding solution. At the near six-leaf stage, leaves and roots of maize seedlings were collected for measurement of relevant indicators and subsequent analysis. Transcriptome sequencing was performed on leaves and roots of the previously identified low phosphorus-tolerant material (CML422) and phosphorus-sensitive material (CIMBL90) under both control (CK) and low phosphorus (LP) treatment conditions, with three biological replicates per sample. Among these, the low phosphorus-tolerant leaves were designated as TCKL and TLPL, and the roots as TCRR and TLPR; the phosphorus-sensitive leaves were labeled as SCKL and SLPL, and the roots as SCRR and SLPR.

### 4.2. Determination of Related Indicators

After washing the vermiculite from the seedling roots, surface moisture was blotted dry using filter paper. Shoot length and root length of the maize seedlings were measured with a ruler. The fresh weights of shoots and roots, as well as the dry weights of shoots and roots, were determined using an analytical balance, and the data were recorded.

### 4.3. Statistical Analysis of Phenotypic Data

Experimental data were organized using Microsoft Excel 2021, descriptive statistics and correlation analysis were performed with IBM SPSS Statistics 26.0, and statistical graphs were generated using Origin 2022.

The calculation formula of generalized heritability [73]:

$$h^{2}=\sigma_{g}^{2}/(\sigma_{g}^{2}+\sigma_{ge}^{2}⁄n+\sigma^{2}⁄nr)$$

Among them, $\sigma_{g}^{2}$ is the genotype variance of the test material, $\sigma_{ge}^{2}$ is the interaction variance between genotype and environment, $\sigma^{2}$ is the error variance, n is the environment number, and r is the repetition number.

### 4.4. Genome-Wide Association (GWAS) Analysis

After filtering all SNP loci, a total of 558,529 SNP markers were obtained with a minor allele frequency (MAF) ≥ 0.05. GWAS analysis was performed using the Mixed Linear Model (MLM) method in Tassel 5.0. The Bonferroni threshold was determined using R(4.3.2), with the significance level set at *p* = 1 × 10^−5^ to identify significant SNPs. Manhattan and Q-Q plots were generated using the “CMplot” package in R.

### 4.5. Gene Analysis of Related Loci

Based on significantly associated SNP markers, candidate genes within 200 kb upstream and downstream regions in the maize reference genome (B73v3) were functionally annotated using the MaizeGDB (https://maizegdb.org/) and NCBI (https://www.ncbi.nlm.nih.gov/) databases.

### 4.6. Transcriptome Analysis

#### 4.6.1. Total RNA Extraction and Illumina Deep Sequencing

Collected samples were rapidly frozen in liquid nitrogen and stored at −80 °C. Total RNA was extracted using the RNA Prep Pure Plant Kit (Tiangen, Beijing, China), and its concentration and purity were measured with a Nanodrop 2000 (Thermo Fisher Scientific, Wilmington, DE, USA). RNA integrity was assessed using the RNA Nano 6000 Assay Kit on the Agilent Bioanalyzer 2100 System (Agilent Technologies, Santa Clara, CA, USA). Qualified samples were used to construct paired-end 150 bp sequencing libraries on the Illumina NovaSeq platform, and sequencing was performed by Biomarker Technologies (Beijing, China).

#### 4.6.2. Quality Assessment of Sequencing Results

Quality control was performed on the raw reads obtained after sequencing. High-quality clean data were obtained by filtering the raw data. The clean reads were then aligned to the Zm_B73_REFERENCE_GRAMENE_5.0 reference genome using Hisat2 (v2.0.4). Following alignment, transcript assembly and gene expression quantification were conducted using StringTie (v2.2.1), and expression levels were normalized using the FPKM (Fragments Per Kilobase of transcript per Million mapped reads) method.

#### 4.6.3. Analysis of Differentially Expressed Genes

Differential gene expression analysis was performed using DESeq2 (v1.30.1). Genes with a fold change ≥1.5 and a *p*-value < 0.05 were considered differentially expressed across all samples. Functional annotation of Gene Ontology (GO) terms was conducted using InterProScan (v5.34-73.0). Additionally, enrichment analysis of differentially expressed genes in KEGG pathways was performed using the KOBAS database and the clusterProfiler(4.4.4) software.

#### 4.6.4. Gene Co-Expression Network Analysis

Weighted Gene Co-expression Network Analysis (WGCNA) is a method for constructing gene co-expression networks. In this study, tools on the Biomarker Cloud Platform were used to process differentially expressed genes obtained from 24 sequencing samples. Genes with FPKM < 1 were removed, with expression threshold set to 1, module similarity threshold to 0.25, and minimum module size to 30 genes. The top 100 to 300 genes by module membership (including duplicates) were used for visualizing the core module gene interaction network with Cytoscape (v3.10.1). Finally, hub genes within key modules were identified based on gene connectivity.

### 4.7. DEG Real-Time Fluorescence Quantitative PCR (RT-qPCR) Validation

Reverse transcription and real-time quantitative PCR were performed using the Evo M-MLV Reverse Transcription Kit from Accurate Biotechnology and the SYBR Green Pro Taq HS Premixed qPCR Kit (with ROX). The reverse transcription conditions were set as follows: 37 °C for 15 min, 85 °C for 5 s, and hold at 4 °C. Primers were designed (Supplementary Table S12) and RT-qPCR was performed on the QuantStudio5 real-time PCR system. The relative expression levels of the selected genes were calculated using the 2^−ΔΔCT^ method, with three biological replicates per treatment [74].

## 5. Conclusions

Under low phosphorus stress conditions, the relevant traits exhibited substantial phenotypic variation and followed an overall normal distribution, indicating that this population is suitable for genome-wide association studies (GWAS). Correlation analysis revealed that the correlation coefficients for most traits reached significant or highly significant levels, further confirming the feasibility of gene mapping through genome-wide association studies (GWAS). In the GWAS analysis, a total of 46 significantly associated QTL loci were detected, encompassing 74 genes, among which 44 have been annotated. Furthermore, a low phosphorus tolerance gene set was constructed using transcriptome sequencing data from both low phosphorus-tolerant and phosphorus-sensitive varieties under normal (CK) and low phosphorus (LP) conditions. Through integration of GWAS and transcriptome analysis results, 18 co-localized genes were identified. Finally, by integrating GWAS analysis, the low phosphorus tolerance gene set, and gene annotation information, 10 candidate genes were identified: Zm00001eb030950, Zm00001eb031210, Zm00001eb031270, Zm00001eb099810, Zm00001eb099820, Zm00001eb136940, Zm00001eb314220, Zm00001eb314230, Zm00001eb364280, and Zm00001eb428910.

## Figures and Tables

**Table 1:** Statistical analysis of traits related to low phosphorus tolerance in maize seedling stage.

Trait	P-Level	Mean ± SD	Rage	CV (%)	G	E	G × E	H^2^	Rd (%)
LL	CK	34.99 ± 7.23	15.17 to 61.5	20.66	**	ns	**	0.85	0.03
	LP	35 ± 7.28	16.7 to 57.75	20.81					
RL	CK	28.67 ± 6.98	11.5 to 51.77	24.33	**	**	**	0.62	3.28
	LP	27.73 ± 7.01	11.1 to 53.85	25.27					
LFW	CK	1.63 ± 0.52	0.4 to 3.28	32.03	**	ns	**	0.80	0.61
	LP	1.62 ± 0.52	0.52 to 3.55	31.96					
RFW	CK	1.06 ± 0.36	0.36 to 2.49	34.43	**	**	**	0.72	5.66
	LP	1 ± 0.36	0.24 to 2.33	36.27					
SDW	CK	0.15 ± 0.09	0.03 to 1.32	60.97	**	ns	**	0.89	6.67
	LP	0.14 ± 0.05	0.04 to 0.33	37.53					
RDW	CK	0.07 ± 0.03	0.02 to 0.26	35.49	**	**	**	0.76	0
	LP	0.07 ± 0.02	0.01 to 0.17	33.93					

Note: LL: seedling length (cm); RL: root length (cm); LFW: aboveground fresh weight (g); RFW: underground fresh weight (g); SDW: aboveground dry weight; RDW: underground dry weight (g); ns: no significant difference; **: *p* < 0.1.

## Data Availability

Data are contained within the article and Supplementary Materials.

## Supplementary Materials

The following supporting information can be downloaded at: https://www.mdpi.com/article/10.3390/plants14182836/s1. Supplementary Table S1: Correlation Analysis of Maize Seedling Traits Under Low Phosphorus Stress; Supplementary Table S2: Significant Single Nucleotide Polymorphisms (SNPs) Associated with Traits Related to Low Phosphorus Tolerance Under Mixed Linear Model (MLM); Supplementary Table S3: Candidate Genes Associated with Low Phosphorus Tolerance Traits in Maize Seedlings Within SNP Intervals; Supplementary Table S4: Quality Control of Sequencing Data; Supplementary Table S5: Reference Gene Alignment Statistics; Supplementary Table S6: Number Statistics of Differentially Expressed Genes (DEGs); Supplementary Table S7: GO Annotation of Differentially Expressed Genes (DEGs) in Maize Seedlings Under Phosphorus Stress; Supplementary Table S8: KEGG Annotation of Differentially Expressed Genes (DEGs) in Maize Seedlings Under Phosphorus Stress; Supplementary Table S9: Seven candidate genes identified through weighted gene co-expression network analysis (WGCNA); Supplementary Table S10: Eighteen genes identified through genome-wide association study (GWAS) and transcriptome analysis; Supplementary Table S11: The 257 maize inbred lines used in the genome-wide association study were propagated in the maize breeding trial field at Huangyang Experimental Station (37.97° N, 102.63° E) in Wuwei City, Gansu Province, China, in 2020. Table S12: Primer sequences for real-time quantitative PCR (RT-qPCR); Supplementary Figure S1: GO bubble plot of differential genes between TCKL and TLPL; Supplementary Figure S2: GO bubble plot of differential genes between TCKR and TLPR; Supplementary Figure S3: GO bubble plot of differential genes between SCKL and SLPL; Supplementary Figure S4: GO bubble plot of differential genes between SCKR and SLPR.
