# Supplementary material for: Identification of Candidate Genes for Low Phosphorus Tolerance in Maize Seedling Stage Based on GWAS and Transcriptome
Source: Plants (Basel). 2025 Sep 11;14(18):2836. doi: 10.3390/plants14182836 (PMC12473607; doi:10.3390/plants14182836)
Supplement: Supplementary file 1 [file plants-14-02836-s001.zip › Figure.pdf]

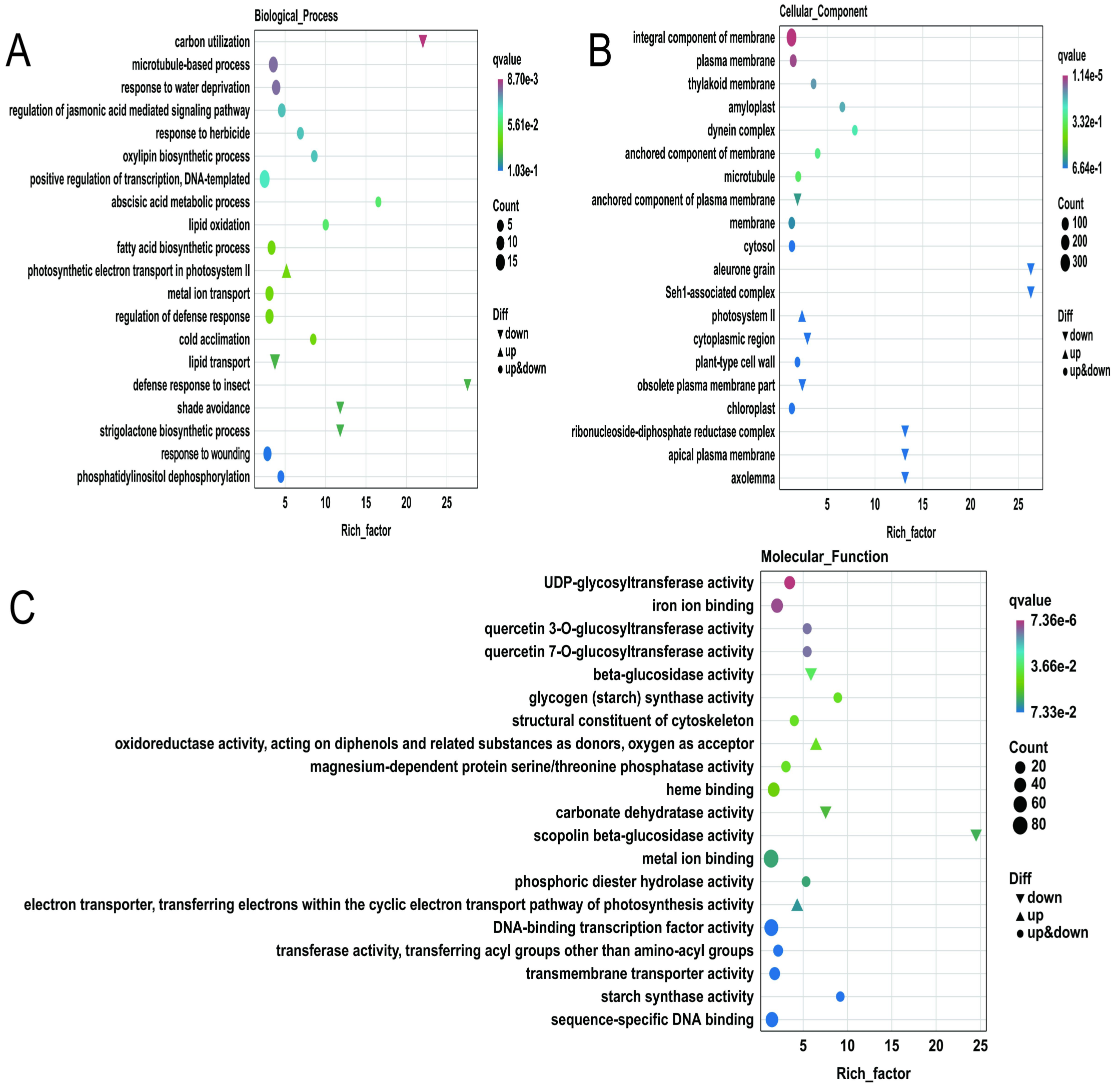

Supplementary Fig. S1. TCKL - vs - TLPL differential gene GO bubble plot

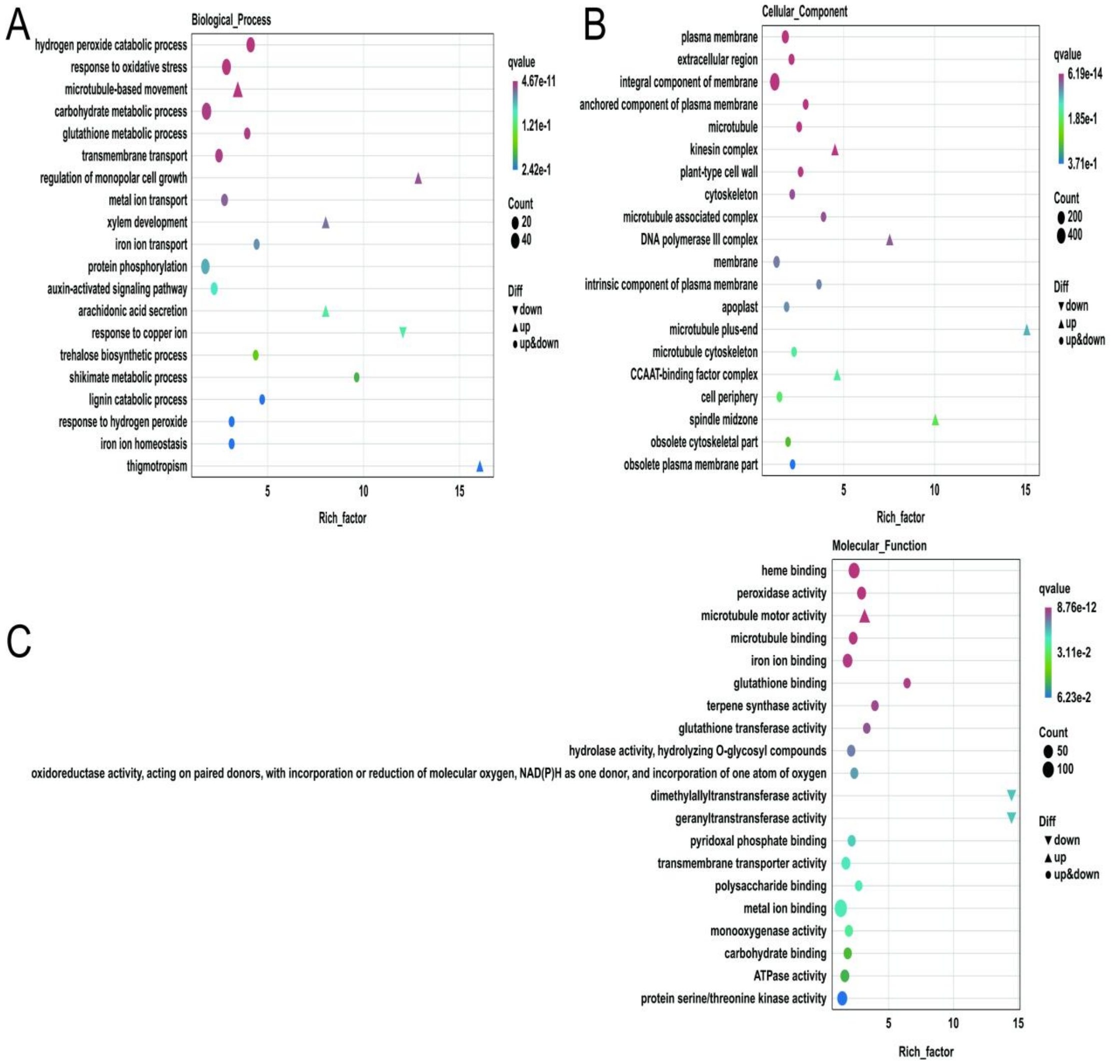

Supplementary Fig. S2. TCKR - vs - TLPR differential gene GO bubble plot

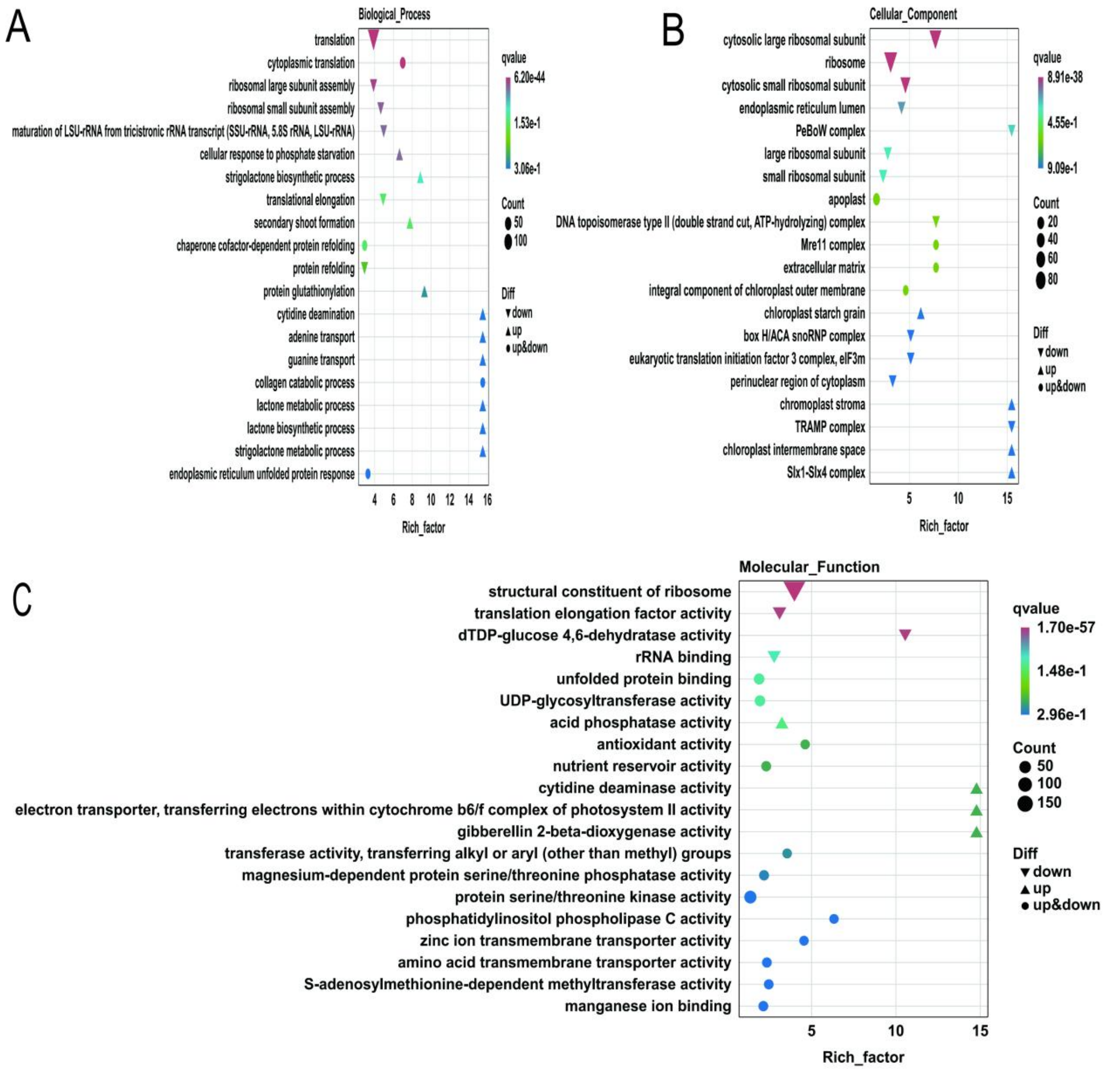

Supplementary Fig. S3. SCKL - vs - SLPL differential gene GO bubble plot

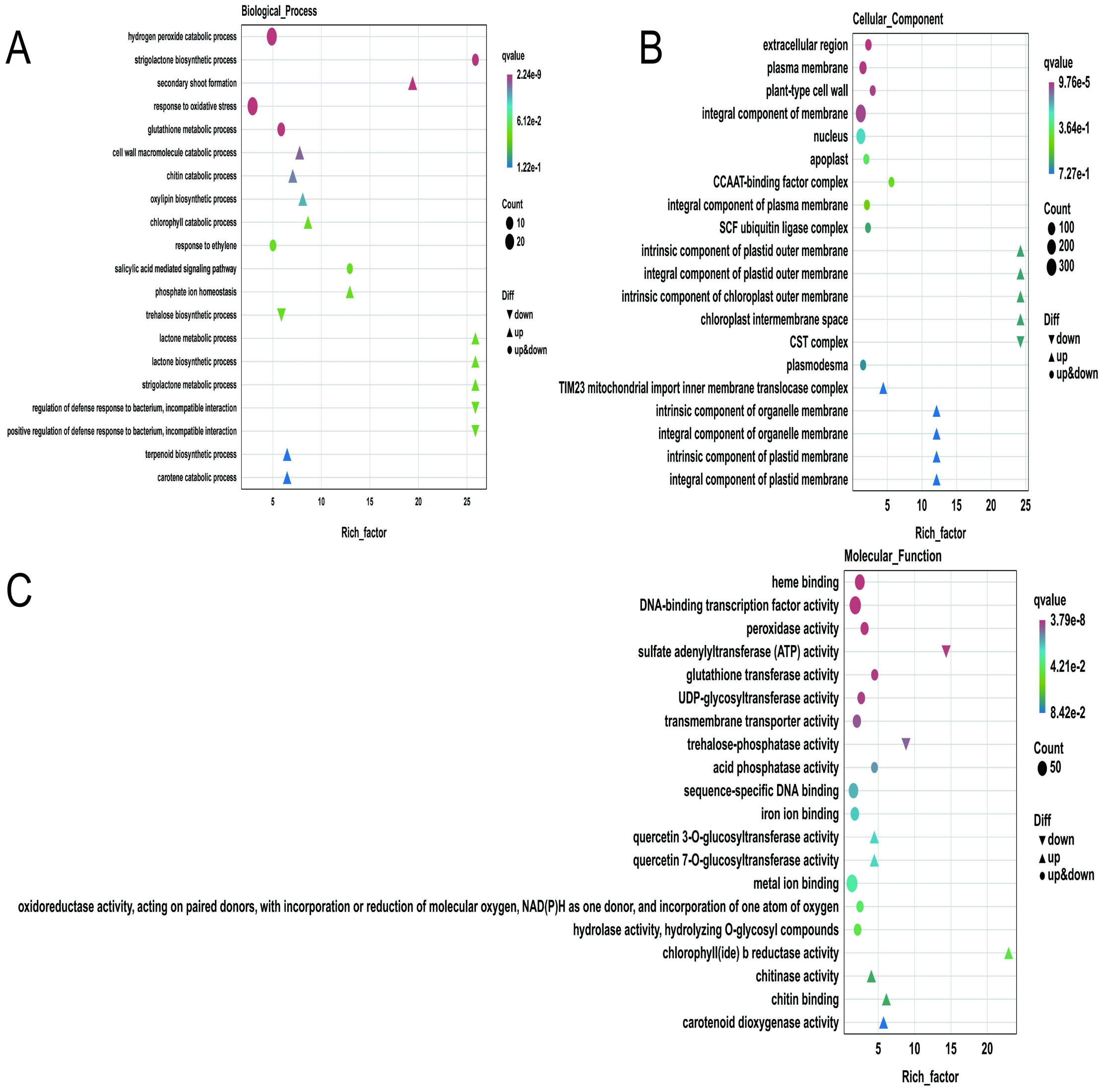

Supplementary Fig. S4. SCKR - vs - SLPR differential gene GO bubble plot
